# Association between internet use and successful aging of older Chinese women: a cross-sectional study

**DOI:** 10.1186/s12877-022-03199-w

**Published:** 2022-06-28

**Authors:** Yao Jiang, Fan Yang

**Affiliations:** 1 Zhou Enlai School of Government, Nankai University, Tianjin, 300350 China; 2 Department of Labor and Social Security, School of Public Administration, Sichuan University, Chengdu, 610065 China

**Keywords:** Successful aging, Internet use, Gender role, Older women, Social exclusion

## Abstract

**Background:**

The internet has become ubiquitous in contemporary human life. However, little is known about the association between internet use and older people’s aging process, especially that of older women.

**Methods:**

Using the nationally representative dataset of the China Longitudinal Aging Social Survey 2016, we examined the relationship between internet usage and the successful aging of older Chinese women. The sample in this study consisted of 2713 respondents with an average age of 69.963 years. Successful aging was defined as no major diseases, no disability, high cognitive functioning, high physical functioning, and active engagement with life. Older women’s internet use behavior was represented by internet use frequency. Probit and instrumental variable models were employed to test the association between internet use frequency and successful aging of older women. The Karlson/Holm/Breen (KHB) mediation analysis was used to estimate the mediating effect of social capital on the relationship between internet use frequency and older women’s successful aging.

**Results:**

Using a probit model (coefficient = 0.030, *p* < 0.001) and an instrumental variable probit model (coefficient = 0.287, *p* < 0.001), it was found that a successful aging status was significantly correlated with an increase in internet use frequency. The functional mechanism analysis suggested that social capital partially mediated the overall association between internet use frequency and successful aging.

**Conclusions:**

This study suggests that the more frequently older Chinese women use the internet, the greater the possibility of successful aging. Our findings provide new evidence from China about the determinants of older women’s aging process and aid in formulating targeted aging policies for older women in developing countries and regions.

## Introduction

During the COVID-19 pandemic, the number of netizens (internet users) aged 60 and older in China doubled, surpassing 123 million [1, 2]. This sharp increase can largely be attributed to the necessity of using the internet to obtain a Health Code (a color-based QR code). To determine an individual’s exposure risk for COVID-19, the Chinese health agencies tracked an individual’s travel history, time spent in high-risk areas, and close contact with potential COVID-19 carriers through the Health Code [3]. When people visit hospitals or other public places, they must show their Health Code. Internet usage is closely related to daily health accessibility, engagement with social activities, and the aging process.

Havighurst proposed the concept of successful aging and defined it as older people obtaining the maximum satisfaction from life [4]. Rowe and Kahn advocated that aging should not primarily be addressed as a process of rapid decline and increasing limitations, thus refining the concept of successful aging [5, 6]. They distinguished successful aging from usual aging and defined successful aging as avoiding disease and disability, high cognitive and physical functioning, and active engagement with life [5, 6], which has been widely used in prior studies [7].

With the internet becoming increasingly ubiquitous in people’s lives, many studies, even those not based on the concept of successful aging, have examined the relationship between internet use and older people’s health outcomes or engagement with life [8–20]. Specifically, regarding health outcomes, a symmetry analysis argued that internet use has a great potential to successfully change the lifestyle of older people aged 50 years or over and promote a healthier body at a very low cost [8]. Prior empirical studies revealed that internet use could provide significant health benefits to older people, including accumulating a wealth of health resources, improving cognitive abilities, and delaying dementia onset [9–12]. Some studies also found that internet use could efficiently decrease depression risk and predict a higher level of subjective well-being and mental health [13–16]. Moreover, regarding engagement with life, some studies found that older netizens had a more active engagement with life, such as involvement in community activities, volunteering activities, and political participation, compared to non-netizens [17–20].

Despite the results in prior literature providing guidelines for exploring the association between internet use and older Chinese people’s aging process, several gaps remain. First, although prior studies have revealed the relationship between internet use and older people’s health or engagement with life, few attempts have been made to examine the relationship between internet use and successful aging and theoretically and empirically explore the potential mediator through which internet use relates to older people’s aging process. Second, gender is a vital social lens [21]. The successful aging of older women and its influencing factors remain under-focused and unexplored in the literature. While women generally have an advantage in lifespan [22], they might experience poverty and ill health in old age due to poor access to education, meaningful work, and health services when younger [23]. Furthermore, approximately two-thirds of the population over the age of 75 in most developing countries comprises women, and an aging society may gradually reflect the feminization of aging [21]. Therefore, it was necessary to integrate a gendered perspective into this research. Third, some quantitative studies have neglected the endogeneity problem between internet use and health or engagement with life, which might produce an estimation bias. In this study, the instrumental variable (IV) approach was used to address the endogeneity problem between internet use and the successful aging of older women.

Therefore, the aims of this study are to clarify the relationship between internet use and the successful aging of women aged 60 years and over in a Chinese setting and to elicit the potential mediator through which internet use is associated with successful aging of older women. To realize the above aims, we used the nationwide dataset of the China Longitudinal Aging Social Survey 2016 (CLASS2016) conducted by Renmin University of China.

This study contributes to the literature in three ways. First, by integrating a gender perspective, this study enriches the literature on the determinants of older women’s aging process by adopting a nationally representative dataset from China. Second, this study investigates the mechanism of social capital through which internet use is associated with the successful aging of older women. In this way, we connected internet use with the successful aging of older women theoretically and empirically. Third, the findings in this study can aid policymakers in formulating targeted aging policies for women in developing countries and regions.

The remainder of the paper is organized as follows. Section 2 presents the theoretical framework and hypotheses. Section 3 provides a description of the CLASS2016 dataset, measurements, and empirical model. Section 4 shows the empirical results. Section 5 includes the discussion and policy implications, and Section 6 states the conclusions.

## Theoretical framework and hypothesis development

### The framework of older women’s successful aging

Successful aging is a multidimensional concept that involves achieving an optimal physical, mental, and social state of well-being for older people [24]. Nonetheless, an agreement on the specific definition and operationalized criteria of successful aging has yet to be reached, and the definition and operationalized criteria of successful aging have varied among studies [7]. Some research has argued that successful aging can be measured in both objective and subjective ways [7, 25]. Objective factors can include older people’s overall health and socioeconomic conditions, while subjective factors include life satisfaction and self-rated physical decline [26].

**Fig. 1:**
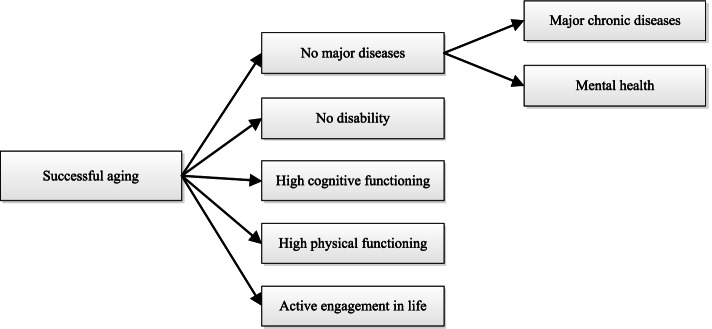
The successful aging framework of the older Chinese women

The most influential conceptualization of successful aging to date was proposed by Rowe and Kahn [27, 28], who defined it as avoidance of disease and disability, high cognitive and physical functional capacity, and an active engagement with life [5, 6]. Among these three specific components, avoidance of disease and disability represents the absence of disease, disability, and related risk factors. High physical and cognitive functional capacities include the potential for an activity. Active engagement with life mainly concerns the engagement forms of interpersonal relations and productive activity [5, 6]. Rowe and Kahn’s conceptualization of successful aging has been used as a “calculable gold standard of aging” in many empirical studies today [28, 29]. Therefore, according to the framework of Rowe and Kahn for successful aging [5, 6] and prior Chinese studies [25, 27, 30], successful aging in the current study has been defined as no major diseases, no disability, high cognitive functioning, high physical functioning, and active engagement with life (Fig. 1).

### Internet use, successful aging, and the mediation effect of social capital

Social capital contributes vital instrumental and emotional resources to the successful aging of older women [31]. Social capital is generally viewed as a resource that can be obtained from individual social networks, and it explains how positive social networks advance individual development by allowing a person to believe that he or she is cared for, loved, esteemed, and valued [32–35]. Prior studies have mainly employed the size of supportive social networks to measure individuals’ social capital, and results have shown that the higher the level of individual social capital, the more likely the individual is to obtain more instrumental resources, such as wealth and medical resources, to prevent disease and maintain good mental health [36–39]. Additionally, a higher level of social capital also provides emotional resources, such as increasing person-to-person contact and extending the friend range so as to promote active social interactions and engagement with life [40, 41]. Thus, a higher level of social capital is conducive to the realization of successful aging. In the current study, we also obtained the social capital of older women through the size of their supportive social networks [36–39].

Moreover, empirical studies have found that, compared with older men, social capital has stronger positive associations with older women’s health and engagement with life [42–44]. This difference can be attributed to the fact that compared to men, women are more willing to spend time preserving and promoting their existing positive social networks among friends and families. Ultimately, women tend to obtain more instrumental and emotional resources from their social capital than men, allowing them to maintain good health and actively engage with life [45]. Thus, social capital is an essential resource for older women to have in order to achieve successful aging.

Internet use may be associated with the successful aging of older women through the mediation of social capital. The internet offers convenience, connectivity, and ubiquity and dismantles the restrictions of kinship and geography on social interactions [46]. Thus, internet use efficiently enhances the frequency of contact between older women and younger generations (e.g., younger friends and younger family members who are highly dependent on the internet) and promotes communication among older people [45]. Moreover, internet use provides users with opportunities to make new friends and expand their social networks [47]. Therefore, internet use can increase social capital among older women. As illustrated by the above discussion, social capital may play a mediating role in the overall association between internet use and the successful aging of older women. Accordingly, we propose the following hypotheses: internet use is positively associated with successful aging for older Chinese women (Hypothesis 1), and social capital can mediate the relationship between internet use and successful aging for older women (Hypothesis 2).

## Methods

### Data

The data employed in this study are from CLASS2016, a nationally representative dataset focusing on people aged 60 and over in China. The dataset was compiled by the China Survey and Data Center at Renmin University of China. It is a nonprofit survey, and respondents answered anonymously to protect their private information. The main content of CLASS2016 comprises respondents’ personal characteristics (e.g., age, education, health status, marital status, retirement planning, and social attitudes) and family characteristics (e.g., family size and family income). CLASS2016 includes respondents from 28 provincial administrative units in China and serves as a source for studying aging issues in the Chinese context. Samples with invalid answers (including “inapplicable” and “unclear” answers) and missing answers were excluded. Consequently, 2713 valid female observations were included in this study.

### Measurements

#### Explained variable

According to the theoretical framework, we defined successful aging of older women as having no major diseases, no disability, high cognitive functioning, high physical functioning, and active engagement with life (Fig. 1). We operationalized the five specific measures as follows.

No major diseases. Respondents were asked whether they had been diagnosed with any major chronic diseases (e.g., chronic lung disease, diabetes, heart disease, kidney disease, liver disease, stroke, etc.). A measure of mental health was also included. Mental health was measured using a nine-item Center for Epidemiological Studies Depression (CES-D) scale, which was validated in a prior study on older adults’ mental health [48]. The positively worded items in the nine-item CES-D scale were reversed, and all items were summed (the internal Cronbach’s alpha of the CES-D scale used was 0.800). Thus, the nine-item CES-D scale ranged from 0 to 18, and a higher score on the scale indicated more depressive symptoms and worse mental health. Based on prior studies on the mental health of older Chinese people [30, 49], we adopted nine as the cut-off point for the nine-item CES-D scale. Thus, when respondents did not report any major chronic diseases and obtained a score of nine or below on the nine-item CES-D scale, they were regarded as meeting the no major diseases criterion and coded as 1; otherwise, 0.

No disability. Based on previous studies [27, 30], no disability was measured by items on basic activities of daily living (BADLs) and instrumental activities of daily living (IADLs). The BADL items included grooming, dressing, bathing, eating, toileting, getting in and out of bed, and continence. The IADL items include phoning, indoor transferring, financial management, public transport, and shopping. Respondents who had no impairment in any items in the BADLs and IADLs met the no disability criterion and were coded as 1; otherwise, 0.

High cognitive functioning. The cognitive functioning assessment developed by CLASS2016 used 16 questions to test older women’s language, nonverbal memory, verbal memory, and conceptualization ability. The response to each item was coded 1 for a correct answer and 0 for a wrong answer. Thus, cognitive functioning assessment scores ranged from 0 to 16. Respondents whose sum scores were at the median (i.e., eight) or higher were considered to achieve the high cognitive function criterion and were coded as 1; otherwise, 0 [28, 30].

High physical functioning. Respondents who had no difficulty in performing each of the five activities of physical functioning (climbing flights of stairs, walking outdoors, lifting or carrying items weighing 5 kg, doing housework, and stooping, kneeling, or crouching) were regarded as reaching the high physical functioning criterion and were coded as 1; otherwise, 0 [27, 50].

Active engagement with life. Respondents who were currently engaged in either productive activities (engaging in paid work) or non-productive activities (community activities or volunteering activities) were considered to meet the active engagement with life criterion and were coded as 1; otherwise, they were coded as 0 [28, 30, 50].

Successful aging. Using the five measures described above, older Chinese women who met all five criteria were regarded as successful agers and were coded as 1; otherwise, 0.

#### Explanatory variable

Internet use frequency was the key explanatory variable in this study and was measured by the question, “How often did you use the internet (including access to the internet through mobile phones) in the past three months?” The answers were given as a number on a five-point Likert scale ranging from 1 (never) to 5 (very frequently).

#### Mediating variable

As mentioned above, social capital may play a mediating role in the relationship between internet use frequency and successful aging among older women. Therefore, social capital was the mediating variable.

Social capital was assessed as a continuous variable, and it was obtained through the total size of the supportive social network, including the number of family members/friends an older woman meets or contacts, the number of family members/friends with whom older woman can confidently discuss private affairs, and the number of family members/friends who can provide help when older woman needs [36–39].

#### Control variables

To precisely clarify the relationship between internet use frequency and the successful aging of older women, we controlled for variables that may affect successful aging. These control variables included older women’s age (years old), marital status (married = 1; otherwise = 0), education (years of schooling), household registration (urban = 1; rural = 0), number of children, living environment (elevator, bathroom, indoor toilet, or flushing toilet in the house = 1; otherwise = 0), industry (respondent engaged in nonagricultural industries before retirement = 1; otherwise = 0), logarithm of total income (including salary, pension, subsidies, rental income from houses and land, and financial support from relatives in 2015), and lifestyle of regular exercise (having the habit of regular exercise = 1; otherwise = 0) [51–56]. In addition, we controlled for regional effects by province. According to the regional division proposed by the National Bureau of Statistics of China [57], the provinces where respondents lived were divided into four categories: eastern, central, western, and northeast regions. Thus, the province was added to the model as a categorical variable. A descriptive analysis of the aforementioned variables is presented in Table 1.

**Table 1:** Descriptive statistics

Variable	Mean	Std. Dev.	Min	Max
Explained variable				
Successful aging	0.173	0.379	0	1
No major diseases	0.401	0.490	0	1
No disability	0.699	0.460	0	1
High cognitive functioning	0.927	0.260	0	1
High physical functioning	0.804	0.397	0	1
Active engagement with life	0.588	0.492	0	1
Explanatory variables				
Internet use frequency	1.415	0.970	1	5
Control variables			0	1
Age	69.963	7.463	60	103
Marital status	0.619	0.486	0	1
Education	6.467	2.843	0	15
Household registration	0.590	0.492	0	1
Number of children	1.381	1.011	0	7
Living environment	0.778	0.416	0	1
Industry	0.511	0.500	0	1
Logarithm of total income	9.187	1.424	3.555	12.899
Lifestyle of regular exercise	0.181	0.385	0	1
Mediating variable				
Social capital	14.766	5.830	0	36
Instrumental variable				
Provincial internet penetration rate	0.525	0.129	0.374	0.731
Observations	2713			

### Empirical model

The explained variable of successful aging was binary; thus, the binary probit model was adopted as the baseline model to analyze the relationship between internet use frequency and the successful aging of older women and verify Hypothesis 1. The baseline binary probit model can be expressed as follows:

1 $$\mathrm{S}{\mathrm{uccessful}\ \mathrm{aging}}^{\ast }={\upbeta}_0+{\upbeta}_1\mathrm{Internet}\ \mathrm{use}\ \mathrm{frequency}+\upgamma \mathrm{Z}+\upvarepsilon$$

where Successful aging^∗^ represents older women’s aging status, which is an unobservable latent variable. Internet use frequency is an observable explanatory variable. Z contains a set of control variables. β_0_ indicates the constant, and β_1_ and γ are the parameter vectors to be estimated. ε is a random disturbance term that obeys a normal distribution.

The relationship between the latent variable of Successful aging^∗^ and Successful aging can be expressed as follows:

2 $$\mathrm{Successful}\ \mathrm{aging}=\left\{\begin{array}{c}1, if\ \mathrm{S}{\mathrm{uccessful}\ \mathrm{aging}}^{\ast }>0\\ {}0, if\ \mathrm{S}{\mathrm{uccessful}\ \mathrm{aging}}^{\ast}\le 0\end{array}\right.$$

Although the baseline binary probit model can indicate the association between internet use frequency and the successful aging of older women to a certain extent, endogeneity remains a considerable problem that needs to be solved. First, although most of the factors affecting older women’s successful aging were controlled for, some unobserved confounding factors could simultaneously affect older women’s internet use behavior and successful aging. Second, the baseline binary probit model is prone to measurement error due to social desirability bias [58]. Thus, to minimize the confounding problem and measurement error, we replicated our baseline analysis by using the IV approach, viz., instrumental variable binary probit model (IV-probit) [59]. The IV-probit model can be summarized into two stages [58, 60]. The first stage involves estimating the main predictor (Internet use frequency) by using an instrument and a set of control variables (*Z*) that were employed to estimate Successful aging^∗^ in Equation (1):

3 $$\mathrm{Internet}\ \mathrm{use}\ \mathrm{frequency}={\delta}_0+{\delta}_1\mathrm{Instrument}+\theta Z+\epsilon$$

where Instrument is the instrumental variable. *δ*_0_ is a constant, and *δ*_1_ is the coefficient of the instrumental variable. *θ* represents the coefficients of the control variables. *ϵ* is a random disturbance term.

The second stage involves estimating the latent variable of Successful aging^∗^ using the value of internet use frequency ($\hat{\mathrm{Internet}\ \mathrm{use}\ \mathrm{frequency}}$) that was predicted in the first stage.

4 $$\mathrm{S}{\mathrm{uccessful}\ \mathrm{aging}}^{\ast }={\mu}_0\hat{+{\mu}_1\mathrm{Internet}\ \mathrm{use}\ \mathrm{frequency}}+\eta Z+\sigma$$

where *μ*_0_ is a constant, and *μ*_1_ is the coefficient of $\hat{\mathrm{Internet}\ \mathrm{use}\ \mathrm{frequency}}$. *η* indicates the coefficients for the control variables and *σ* is a random disturbance term. Successful aging^∗^ and *Z* have the same meanings as those in Equation (1).

In this study, the provincial internet penetration rate in 2015 was chosen as the instrument. The provincial internet penetration rate is the number of internet users in the province divided by the total provincial population, which is an appropriate IV satisfying the requirements of relevance and exclusion [61]. Regarding relevance, individual internet use behavior is closely associated with the level of internet penetration in a region. Regarding exclusion, according to the ratchet effect, the internet infrastructure within a region is the result of a long period of construction, which is irreversible and does not directly affect older women’s successful aging. In the Results section, the IV was tested to validate its reasonableness, including the tests of weak identification and under-identification, the Wald test of exogeneity, and the F value of the first stage.

Furthermore, to explore the influential mechanism and demonstrate support for Hypothesis 2, a binary probit model based on the KHB mediation analysis as proposed by Karlson, Hom, and Breen was employed. The KHB mediation method compares the full model (direct effect) with a reduced model (total effect) that substitutes the residuals of the mediator from a regression of the mediator on the key variables [62, 63]. This method extends the decomposition properties of linear models to nonlinear probability models, and enables the separation of the change in the coefficient due to either confounding or rescaling [62, 63]. STATA 15.0 was used to perform the data analyses in the current study (StataCorp. LP., College Station, TX, USA).

## Results

### Descriptive analysis

A descriptive analysis of all the variables is reported in Table 1 (*n* = 2713). Regarding the explained variable, the mean value of successful aging was 0.173 (SD = 0.379), indicating that 17.3% of the older Chinese women in our sample met all five measures of successful aging and were successful agers. Concerning the five specific measures of successful aging, the mean value of no major diseases was 0.401 (SD = 0.490), which suggested that the ratio of participants who met the no major diseases criterion was 40.1%. The measure of no disability had an average value of 0.699 (SD = 0.460), indicating that the proportion of those who met the disability criterion was 69.9%. The mean values of high cognitive and physical functioning were 0.927 (SD = 0.260) and 0.804 (SD = 0.397), respectively. The results indicated that the ratios of participants who met the criterion of high cognitive and high physical functioning were 92.7 and 80.4%, respectively. Finally, the measure of active engagement with life had a mean value of 0.588 (SD = 0.492). The results indicated that 58.8% of the older Chinese women in our sample actively participated in non-productive or productive activities. The explanatory variable of internet use frequency had an average value of 1.415 (SD = 0.970). This result suggested that the older Chinese women in our sample were relatively inactive in using the internet.

In terms of the control variables, the mean age of the sampled older women was 69.963 (SD = 7.463), and 61.9% were married. The average educational attainment of the older respondents was 6.467 years (SD = 2.843), which was approximately the level of primary school. A total of 59% of the sampled women were urban residents, and the average number of children that the sampled women had was 1.381 (SD = 1.011). The mean value of the living environment was 0.778 (SD = 0.416), and of the sampled individuals, 51.1% engaged in nonagricultural industrial work before retirement. The average value of the logarithm of total income was 9.187 (SD = 1.424), and 18.1% of the respondents had a regular exercise habit.

The mean value of the mediating variable social capital was 14.766 (SD = 5.830). The mean value of the IV (i.e., provincial network penetration rate) was 0.525 (SD = 0.129), suggesting that the average provincial network penetration rate for provinces where the sampled older women lived in 2015 was 52.5%.

### Results of the baseline regression and instrumental variable regression

Table 2 presents the results of the baseline and IV regressions. As shown by the baseline binary probit model results in Table 2, the coefficient of internet use frequency is significant and positive (coefficient = 0.030, *p* < 0.001). This indicates that, with other factors being certain, an increase in internet use frequency is associated with an increase in the probability of being in the successful aging status by three percentage points. The coefficient of internet use frequency is again significant and positive (coefficient = 0.287, *p* < 0.001) when employing the IV-probit model, suggesting that an increase in internet use frequency is correlated with an increase in the probability of being in a successful aging status by approximately 29 percentage points. The above results confirm Hypothesis 1—that the higher the internet use frequency, the more likely older Chinese women are to be in a successful aging status. In addition, the coefficient of internet use frequency, estimated using the IV-probit model, is approximately ten times higher than that estimated using the baseline binary probit model. This underestimation can be attributed to the fact that the baseline binary probit model considerably underestimates the effect of internet use frequency due to confounding problems and measurement errors [58, 64, 65].

**Table 2:** The relationship between internet use frequency and successful aging of older Chinese women

Variable	Probit	IV-probit
Internet use frequency	0.030***	0.287***
	(0.008)	(0.066)
Age	−0.001	0.003
	(0.001)	(0.002)
Marital status	0.007	0.047*
	(0.015)	(0.019)
Education	0.007*	−0.021**
	(0.003)	(0.008)
Household registration	0.062**	0.077***
	(0.020)	(0.022)
Number of children	−0.005	0.006
	(0.008)	(0.009)
Living environment	0.080***	0.059**
	(0.023)	(0.021)
Industry	−0.015	− 0.003
	(0.019)	(0.022)
Logarithm of total income	0.009	−0.007
	(0.006)	(0.008)
Lifestyle of regular exercise	−0.071***	− 0.101***
	(0.020)	(0.018)
Social capital	0.005***	0.002
	(0.001)	(0.002)
Central region	0.110***	0.288***
	(0.020)	(0.057)
Western region	0.092***	0.315***
	(0.022)	(0.070)
Northeast region	−0.082***	0.034
	(0.016)	(0.052)
Pseudo R^2^	0.092	–
**IV tests**		
Weak IV identification test	Cragg-Donald Wald F statistic	65.432
	Kleibergen-Paap rk Wald F statistic	77.227
Under-identification test	Kleibergen-Paap rk LM	65.951
First-stage F statistic	77.230***	
Wald test of exogeneity (*χ* ^2^)	23.350***	
Observations	2713	

The estimates represent marginal effects; Standard errors in parentheses; IV represents instrumental variable; *** *p* < 0.001, ** *p* < 0.01, * *p* < 0.05

Table 2 also reports the reasonableness of the IV. First, the Cragg-Donald Wald F statistic and Kleibergen-Paap rk Wald F statistic are 65.432 and 77.227, respectively. These statistics are significantly higher than the Stock-Yogo weak IV test critical value of 16.38 (10% maximal IV size) [66]. Therefore, the null hypothesis that the provincial internet penetration rate is a weak IV is rejected. Second, the Kleibergen-Paap rk LM statistic is 65.951 (*p* < 0.001), and the first-stage F statistic is 77.230 (*p* < 0.001). The above results indicate that IV (i.e., the provincial internet penetration rate) is closely related to the endogenous explanatory variable (internet use frequency). Finally, the result of the Wald test of exogeneity (*χ* ^2^ = 23.350, *p* < 0.001) significantly reject the null assumption that internet use frequency is an exogenous variable, which means that the IV-probit results should be chosen over the results of the baseline binary probit, which might suffer from confounding problems and measurement errors. The above results of the IV tests provide evidence that the IV (i.e., the provincial internet penetration rate) is valid, and the estimated equations are correctly specified.

### Robustness checks

#### Subgroup regression

The full sample was categorized into rural and urban samples based on household registration. The respondents were divided into two subgroups according to marital status: married and other. Considering the industry in which respondents engaged before retirement, the sampled older women were placed into two subgroups: agricultural and nonagricultural. The results of the six subgroups regressions using the IV-probit models are presented in Table 3. The results show that the coefficients of internet use frequency in the rural (coefficient = 0.268, *p* < 0.001) and urban groups (coefficient = 0.440, *p* < 0.01), and those in the married (coefficient = 0.295, *p* < 0.001) and other (coefficient = 0.310, *p* < 0.01) groups, are positive and significant. In addition, the coefficients of internet use frequency in the agricultural (coefficient = 0.233, *p* < 0.01) and non-agricultural (coefficient = 0.599, *p* < 0.05) groups are also positive and significant. Thus, the results that internet use is positively associated with successful aging for older Chinese women are valid across different subgroups.

**Table 3:** Robustness check of subgroup regression (IV-probit)

Variable	Household registration		Marital status		Industry	
	Rural	Urban	Married	Other	Agricultural	Non-agricultural
Internet use frequency	0.268***	0.440**	0.295***	0.310**	0.233**	0.599*
	(0.074)	(0.158)	(0.089)	(0.112)	(0.075)	(0.264)
Age	0.002	0.006	0.004	0.001	−0.000	0.012
	(0.002)	(0.004)	(0.003)	(0.002)	(0.002)	(0.007)
Marital status	0.078**	0.021	–	–	0.043	0.056
	(0.026)	(0.029)	–	–	(0.025)	(0.040)
Education	−0.040**	−0.011	− 0.010	− 0.034	−0.027*	− 0.023
	(0.014)	(0.008)	(0.006)	(0.018)	(0.013)	(0.015)
Household registration	–	–	0.019	0.152***	0.107**	−0.083
	–	–	(0.031)	(0.042)	(0.034)	(0.108)
Number of children	−0.004	0.008	0.012	−0.007	−0.006	0.015
	(0.011)	(0.015)	(0.012)	(0.016)	(0.011)	(0.020)
Living environment	0.025	0.077	0.084**	0.029	0.053*	0.070
	(0.023)	(0.045)	(0.026)	(0.037)	(0.024)	(0.055)
Industry	0.032	−0.052	−0.023	0.007	–	–
	(0.042)	(0.040)	(0.028)	(0.040)	–	–
Logarithm of total income	−0.002	− 0.025	0.002	− 0.021	−0.007	− 0.011
	(0.009)	(0.016)	(0.010)	(0.014)	(0.009)	(0.020)
Lifestyle of regular exercise	−0.061**	−0.145***	− 0.117***	−0.070*	− 0.056*	−0.168***
	(0.022)	(0.032)	(0.023)	(0.030)	(0.025)	(0.043)
Social capital	0.003*	−0.005	−0.000	0.004	0.003	−0.010
	(0.002)	(0.004)	(0.002)	(0.003)	(0.002)	(0.009)
Central province	0.055	0.567***	0.261***	0.367***	0.138**	0.669**
	(0.037)	(0.143)	(0.069)	(0.109)	(0.043)	(0.218)
Western province	0.145*	0.519***	0.283***	0.416**	0.150**	0.748***
	(0.059)	(0.153)	(0.085)	(0.134)	(0.051)	(0.203)
Northeast province	−0.060	0.183	0.011	0.102	−0.025	0.331
	(0.031)	(0.146)	(0.063)	(0.103)	(0.039)	(0.282)
Observations	1112	1601	1678	1035	1328	1385

The estimates represent marginal effects; Standard errors in parentheses; *** *p* < 0.001, ** *p* < 0.01, * *p* < 0.05

#### Testing the relationship between internet use frequency and the five specific measures

As the five specific measures of successful aging were binary, using the IV-probit models to estimate the relationship between internet use frequency and the five specific measures of successful aging is appropriate. The specific procedure of IV-probit models estimating the five specific measures is the same as that used to estimate successful aging. The results are estimated using the IV probit model, as presented in Table 4. The results show that internet use frequency is positively and significantly correlated with no major diseases (coefficient = 0.299, *p* < 0.001), no disability (coefficient = 0.163, *p* < 0.05), high cognitive functioning (coefficient = 0.140, *p* < 0.001), high physical functioning (coefficient = 0.299, *p* < 0.001), and active engagement with life (coefficient = 0.225, *p* < 0.01). These results indicate that the Hypothesis 1 of our study is maintained again.

**Table 4:** Robustness check of testing the relationship between internet use frequency and five specific measures of successful aging (IV-probit)

Variable	No major diseases	No disability	High cognitive functioning	High physical functioning	Active engagement with life
Internet use frequency	0.299***	0.163*	0.140***	0.299***	0.225**
	(0.085)	(0.071)	(0.031)	(0.068)	(0.081)
Age	0.009***	−0.012***	−0.001	−0.003*	− 0.000
	(0.002)	(0.002)	(0.001)	(0.002)	(0.002)
Marital status	0.069**	0.054*	0.033**	0.087***	−0.029
	(0.026)	(0.023)	(0.012)	(0.023)	(0.026)
Education	−0.026**	0.000	−0.008*	−0.023**	− 0.013
	(0.010)	(0.008)	(0.004)	(0.008)	(0.010)
Household registration	0.082**	0.042	0.044**	0.047	0.048
	(0.031)	(0.027)	(0.014)	(0.026)	(0.030)
Number of children	0.007	−0.021*	0.005	−0.004	0.015
	(0.012)	(0.010)	(0.004)	(0.009)	(0.012)
Living environment	0.051	0.013	−0.013	0.014	0.052
	(0.030)	(0.025)	(0.009)	(0.024)	(0.030)
Industry	0.015	−0.002	0.026*	−0.009	− 0.060*
	(0.029)	(0.025)	(0.012)	(0.024)	(0.028)
Logarithm of total income	−0.022*	0.009	−0.003	0.010	0.000
	(0.011)	(0.009)	(0.004)	(0.009)	(0.011)
Lifestyle of regular exercise	−0.108***	−0.042	0.002	−0.112***	− 0.055
	(0.031)	(0.031)	(0.015)	(0.033)	(0.033)
Social capital	0.001	0.003	0.000	−0.003*	0.005**
	(0.002)	(0.002)	(0.001)	(0.002)	(0.002)
Central province	0.176***	0.112**	0.055***	0.205***	0.248***
	(0.053)	(0.039)	(0.012)	(0.027)	(0.044)
Western province	0.186**	0.161***	0.051***	0.172***	0.259***
	(0.059)	(0.038)	(0.011)	(0.027)	(0.045)
Northeast province	0.079	0.044	0.054***	0.124***	−0.093
	(0.058)	(0.044)	(0.009)	(0.030)	(0.055)
Observations	2713				

The estimates represent marginal effects; Standard errors in parentheses; *** *p* < 0.001, ** *p* < 0.01, * *p* < 0.05

### Functional mechanism analysis

After the positive association between internet use frequency and successful aging of older women was verified, the potential mechanism through which internet use frequency was correlated with successful aging of older women was tested. The results of binary probit model based on the KHB mediation test on social capital are presented in Table 5. The results in Table 5 show that, the total and direct effects of internet use frequency on older women’s successful aging were 0.140 (*p* < 0.001) and 0.129 (*p* < 0.001), respectively, and the indirect effect of internet use frequency was 0.011 (*p* < 0.01) with 7.83% of the total effect being mediated by social capital.

**Table 5:** Mediating effect of social capital using KHB mediation analysis

	Successful aging
Internet use frequency	
Reduced (total effect)	0.140***
	(0.035)
Full (direct effect)	0.129***
	(0.035)
Indirect effect (mediating effect of social capital)	0.011**
	(0.004)
Confounding percentage	7.83%
Observations	2713

Controls: age, marital status, education, household registration, number of children, living environment, industry, logarithm of total income, lifestyle of regular exercise, and province; Standard errors in parentheses; *** *p* < 0.001, ** *p* < 0.01

## Discussion

During the COVID-19 pandemic, the internet-based Health Code became a major part of people’s lives and is a vivid and incisive reflection of the influence of the internet on older people in China. When older people take public transport (e.g., bus, subway, and train), access public places, and migrate across provincial boundaries, they must show their Health Code. As a result, the number of netizens aged 60 years and older in China surged in 2020 [1, 2]. Internet use has become an important part of the aging process of older people. In this context, it is of practical significance to explore the relationship between internet use and aging.

Previous studies have focused on the association between internet access and a single aspect of older people’s aging, such as health outcomes or engagement with life [10–16]. However, this study investigated the relationship between internet use frequency and multidimensional components of successful aging among older Chinese women, based on Rowe and Kahn’s framework of successful aging. The results found a positive relationship between internet use frequency and successful aging of older Chinese women.

The results further indicated that social capital partially mediated the overall association between internet use frequency and successful aging. Internet usage shortens the geographical and social distances among individuals and enables older women to communicate with their children, relatives, and friends thousands of miles away [67, 68]. In particular, the voice messaging function of internet-based applications, such as the voice chats in WeChat and QQ, greatly promotes communication between older people and the younger generation, advances the removal of barriers posed by age segregation, and shrinks the gaps between generations [69]. Furthermore, internet usage broadens the ability of older women to acquire health-related knowledge, self-display, strengthen social connections, expand social capital, and consequently achieve a successful aging process. As of April 2021, TikTok creators aged 60 and over had made more than 600 million videos on the platform, with content including the display of lifestyles and interactions between parents and children, which had received over 40 billion “likes” in total [70].

Based on the findings of this study, active usage of the internet is one of the keys to achieving successful aging of older women. Therefore, some policy recommendations based on internet use can be proposed to provide external support for the successful aging of older women. First, older people’s internet use difficulties still deserve our attention, especially among older women who have historically been denied access to educational resources in some developing countries and regions. To meet most of the needs of older women in using the internet, suppliers of digital devices should try to design smartphones or laptops that are affordable, easy to carry, easy to access the internet, rich in functions, and especially easy to operate.

Second, because a sustained preference for sons and discrimination against daughters has been more efficiently maintained in some undeveloped rural areas, older women in rural areas are less educated and have more vulnerable livelihoods than men and urban women. In the subgroup regression, we found that internet use frequency is significantly and positively associated with successful aging of both urban and rural older women. Thus, to advance the successful aging of these rural older women, elderly care services or poverty alleviation measures of relevant departments can consider subsidizing the purchase of smartphones, and recruiting volunteers to teach them how to use smartphones to facilitate internet usage for older women in undeveloped rural areas.

Third, women could be more easily addicted to social networking services than men [71]. While internet use is largely beneficial for older women’s successful aging, there is an increasing number of female senior citizens with smartphone addiction, such as long-term TikTok browsing and overuse of multimedia applications. Moreover, women, especially older women, can be victims of online scams [72, 73]. Therefore, the government and relevant institutions can guide older women in appropriate internet usage to prevent internet addiction and improve their awareness of internet fraud.

This study has limitations that should be considered. First, considering that internet overuse may result in adverse effects (e.g., dizziness and hypertension) on older people, we tested the inverse U-shaped relationship between the internet use frequency and successful aging of older women. The results of the U-test indicated that the extreme point was not included in the range of the explanatory variable, thereby failing to reject the null assumption of monotonicity. However, the monotonic relationship between internet use frequency and older people’s successful aging needs to be further examined.

Second, the data used in this study were cross-sectional rather than panel data; thus, we could not observe dynamic changes in the relationship between internet use frequency and successful aging of older women. In the future, research can employ panel data to test this.

Third, older women can use the internet either adaptively or maladaptively. However, due to data availability restrictions, we cannot identify older people who use the internet in adaptive or maladaptive ways in CLASS2016. Future research could explore the different effects of adaptive and maladaptive methods of internet use on older women’s aging processes.

Fourth, restricted by data availability, we only reported the mediating effect of social capital on the relationship between internet use frequency and successful aging among older women, while internet use is associated with multiple purposes beyond social communications. Thus, social capital is not the only possible mediator. Future studies can explore other potential mediators.

## Conclusion

The internet has become almost ubiquitous in all aspects of contemporary human life. However, little is known about the association between such profound technological developments and older people’s aging, especially older women. This study offers preliminary evidence that internet use is associated with the aging process of older women in China. Based on the framework of successful aging proposed by Rowe and Kahn, we used a nationwide dataset and found that the positive association between internet use frequency and the successful aging of Chinese women aged 60 and over was valid across different models. Moreover, social capital partially mediated the relationship between internet use frequency and successful aging of older Chinese women. The findings enrich the literature on the determinants of successful aging of older women and aid policymakers in formulating targeted aging policies for older women in developing countries and regions.

## Data Availability

Restrictions apply to the availability of these data. Data was obtained from: http://class.ruc.edu.cn.

## Abbreviations

IV: Instrumental variable
CLASS2016: China Longitudinal Aging Social Survey
CES-D: Center for Epidemiological Studies Depression
IV-probit: Instrumental variable binary probit
